# Impact of Farm Management Practices on Tick Infestation in Punjab’s Livestock: A Comprehensive Epidemiological Study

**DOI:** 10.3390/ani14162437

**Published:** 2024-08-22

**Authors:** Muhammad Husnain Ali Alvi, Abdul Rehman, Tariq Jamil, Muhammad Zahid Iqbal, Aneela Zameer Durrani, Aman Ullah Khan, Muhammad Usman, Carola Sauter-Louis, Franz J. Conraths

**Affiliations:** 1 Institute of Epidemiology, Friedrich-Loeffler-Institut, 17493 Greifswald-Insel Riems, Germany; carola.sauter-louis@fli.de (C.S.-L.); franz.conraths@fli.de (F.J.C.); 2 Department of Epidemiology and Public Health, University of Veterinary and Animal Sciences, Lahore 54100, Pakistan; abdul.rehman@uvas.edu.pk; 3 Institute of Bacterial Infections and Zoonoses, Friedrich-Loeffler-Institut, 07743 Jena, Germany; tariq.jamil@fli.de; 4 Department of Veterinary Medicine, University of Veterinary and Animal Sciences, Lahore 54100, Pakistan; zahid.iqbal@uvas.edu.pk (M.Z.I.); aneela@uvas.edu.pk (A.Z.D.); muhammad.osman@uvas.edu.pk (M.U.); 5 Section of Microbiology, Department of Pathobiology, College of Veterinary and Animal Sciences, Sub-Campus (University of Veterinary and Animal Sciences, Lahore), 12 Km Chiniot Road, Jhang 35200, Pakistan; amanullah.khan@uvas.edu.pk

**Keywords:** epidemiology, ruminants, tick infestation, farm management, tick control, Pakistan

## Abstract

**Simple Summary:**

This cross-sectional study evaluated management factors contributing to tick infestation in 96 livestock farms in Punjab, Pakistan. Data collected via in-person interviews and statistical analysis revealed soft bedding as a significant risk factor. Other factors included lack of quarantine, improper drainage, infrequent veterinary visits and hot seasons. Strict quarantine practices, hard bedding, improved farm structure and a combination of tick control remedies were emphasized. Collaboration among farmers, veterinarians and researchers was deemed essential.

**Abstract:**

Tick infestation poses an important challenge to livestock in Pakistan. Farm management practices and environmental variables can influence tick infestation prevalence in animals. To this end, a cross-sectional survey of 96 farms in four different geographical districts (24 farms from each district) was conducted in Punjab, Pakistan, between October 2021 and January 2022. An epidemiological questionnaire was designed focusing on farm management practices and their impact on tick infestations at these farms. Data were collected via in-person interviews. Regional and farm-specific variables’ associations were evaluated using Pearson’s chi-square test and Fischer’s exact test, respectively. A multivariable logistic regression model was used to identify significant risk factors. This study identified that using soft bedding materials, e.g., wheat straw, leaf litter or plain soil posed a significant risk of tick infestation. Additionally, the absence of quarantine measures, open sheds and inadequate drainage were found as contributing factors in univariable analysis. Higher tick prevalence in the hotter seasons highlighted the influence of Punjab’s extreme weather on tick infestation. Despite regular veterinary visits and the use of acaricidal drugs, the prevalence of tick infestation at these farms suggested potential drug resistance in the ticks. The study recommended establishing quarantine practices, improving farms’ drainage systems and bedding and using a combination of chemical and traditional remedies to tackle drug resistance in ticks. Education and awareness programs on tick-borne diseases and control measures are advocated to reduce the tick infestation burden on animals. Further research on longitudinal studies to better understand tick population dynamics and develop effective acaricides is encouraged. This called for collaborative control efforts among farmers, veterinarians and research institutions.

## 1. Introduction

Livestock industry plays an important role in Pakistan’s agriculture-based economy, where owning livestock is a symbol of the social status of the owner and a ready source of income [1,2]. The livestock sub-sector contributed to 14.63% of the national Gross Domestic Product (GDP) and 60.84% to the Agriculture Value Addition (AVA) in the fiscal year 2024 [3]. Moreover, it directly employed more than 8 million rural families for their livelihood, where cattle, water buffaloes, goats and sheep contributed mainly to the total estimated livestock population of 223.5 million in 2024 [3]. Pakistan is located in the sub-tropical zone of South Asia, which has a significant influence on agriculture and livestock farming practices, especially the occurrence of diseases in farm animals [4,5]. Ectoparasites such as ticks, are notable to transmit pathogens that cause viral, bacterial and protozoal diseases, thereby posing a significant health risk to the livestock sector [6,7].

Ticks belong to the class Arachnida and are hematophagous arthropods that feed on the blood of animals and humans [8]. They often infest specific areas on the host, e.g., the udder, ear, neck, tail and brisket region of the animals, known as predilection sites. Ticks serve as reservoirs for various pathogens due to their potential symbiotic relationship with these pathogens [9], thus acting as vectors in disease transmission. In Pakistan, tick-borne diseases commonly prevail in livestock species, e.g., cattle, buffaloes, goats and sheep. These diseases include babesiosis, theileriosis and anaplasmosis caused by protozoa such as *Babesia (B.) bovis, B. bigemina* and *Anaplasma (A.) marginale, Theileria (T.) ovis* and *T. lestoquardi* [10,11]. Studies have reported the prevalence of ticks (*Hyalomma* spp., *Rhipicephalus* spp. and *Haemaphysalis* spp.) in Pakistan, that transmit these diseases [10,12]. Moreover, ticks can transmit other various bacterial and viral zoonotic diseases such as Lyme disease, rickettsiosis, Crimean–Congo Hemorrhagic Fever (CCHF) and tick-borne encephalitis, making them of significant public health importance [9,13,14].

Punjab is the most populated and economically developed province of Pakistan. To the best of the authors’ knowledge, it has a human population of 127.69 million, in 41 districts, as of 2023 [15,16]. In addition, it is home to the largest number of agricultural, livestock and poultry farms in the country. The climate conditions in Punjab favor tick infestation in livestock, especially in the summer season, due to hot and humid conditions, leading to economic losses due to reduced production and fertility [8,17]. Despite various control measures taken against ticks, many of them have proven to be ineffective mainly due to acquired resistance in many species. Moreover, the commonly practiced anti-tick therapy in livestock species is often not considered cost-effective for small- or medium-sized livestock farms due to unsatisfactory efficacy and unaffordable costs of the acaricides [18]. The rapid adaptation of ticks to the habitats in Pakistan, supported by the climatic conditions in the country, remains a persisting challenge. Thus, the present survey aimed to determine the relationship among various farm attributes, risk factors and tick control measures in Punjab’s livestock management systems. It also evaluated the knowledge and awareness of livestock owners regarding farm management, ticks and tick-borne diseases.

## 2. Materials and Methods

### 2.1. Study Area

The survey was conducted in four districts of Punjab, i.e., Lahore, Kasur, Chakwal and Bahawalpur. These districts were selected based on their livestock population, livestock marketing and their location in different agro-ecological zones, i.e., mainly the arid and semi-arid zones [19]. The district Lahore is located at 31.52° N, 74.35° E on the bank of the river Ravi, with an average temperature from 43 °C in summer to 9 °C in winter. The average relative humidity is 60% in summer and 35% in winter with an average annual rainfall of 150 mm. Large herds of livestock, especially buffaloes, prevail along the bank of the river Ravi and outside the metropolitan area of the district Lahore. The district Kasur is located at 31.11° N, 74.44° E, sharing a boundary with the district Lahore. It has an average temperature of 40 °C in summer and 6.5 °C during winter. The average annual rainfall is 170 mm with an average humidity of 60% annually. District Kasur is rich in livestock and milk production supported by the fertile agricultural lands along the rivers Ravi and Sutlej. Both Lahore and Kasur districts fall under the arid to semi-arid agro-ecological zone of Pakistan.

Chakwal is located at 32.91° N, 72.85° E, in the northern rangelands of Punjab. It is bordered by the districts Jehlum to the east, Rawalpindi and Attock to the north, Mianwali to the west and Khushab to the south extending into Salt Range. Its average temperature in summer is 34 °C, and in winter, it is 13 °C, with an annual rainfall of 114 mm. The average relative humidity level is 33.3% annually. Chakwal is dominated by a mixed livestock farming system, predominantly comprising cattle, buffaloes, sheep and goats. The region is mostly based on agro-pastoral livelihood, where livestock plays a crucial source of ready income.

The Bahawalpur district is located at 30.53° N, 71.78° E on the bank of river Sutlej, in the south of the Punjab. This district is bordered by India in the south and the southeast, districts Bahawalnagar, Vehari, Lodhran, Multan and Muzaffargarh to the north and district Rahimyar Khan to the west. Approximately, two-thirds of the district is covered by the Cholistan desert which merges into the Thar desert. District Bahawalpur has a maximum average temperature of 37.5 °C in summer and a minimum of 8 °C in winter, and much less annual rainfall (143 mm). The average relative humidity level varies between 28.62–59.92% [20]. Animal rearing forms the backbone of livelihood in this region. Large herds of cattle, camels, sheep and goats prevail in this region. Notable breeds include Cholistani cattle, Beetal and Nachi goats, Buchi, Sipli and Cholistani (Khadali) sheep and Marrecha and Barella camels [21].

### 2.2. Study Design

The sample size for farms was calculated assuming 50% tick infestation prevalence at farm levels, a confidence level of 95% and a desired precision of 10% for the prevalence estimate [22]. Thus, a total of 96 farms, rearing four main livestock species (cattle, buffaloes, goats and sheep) and distributed evenly among the selected districts, were randomly chosen. Only mixed farms with at least two animals of each of the four livestock species, i.e., cattle, buffaloes, sheep and goats, were selected, resulting in a total herd size of a minimum of eight ruminants [19]. Farms from each district were selected randomly using a random number generator in an Excel file (Microsoft Office, Excel 365, Redmond, WA, USA) sheet using the RAND() function. All selected farms were located in rural and semi-urban areas of the districts. A farm was assumed to have a high tick infestation if at least five ticks were found on the sampled animals of each species at the farm. The animals were inspected visually to identify the predilection sites of the ticks, e.g., ears, around the neck and chest and dewlap for cattle, shoulder blades, udder region in females and around the testicles in males and the perineum region and tail as mentioned previously [19]. The tick burden on each animal was counted by the number of ticks present on the body. Additional information was recorded on a predesigned epidemiological questionnaire (Supplementary Materials). The survey was conducted between October 2021 and January 2022.

### 2.3. Data Collection

A questionnaire was designed based on plausible factors for tick infestation identified in various studies [13,14,19,23]. Farm owners and workers were interviewed in person during farm visits using national (Urdu) or local language (Punjabi) to fill the questionnaire. The farmers responded based on their free will following informed verbal consent. The questionnaire included information on characteristics of the sampled animal population and farm management practices, such as the farm area, farm boundary, waste disposal, and drainage system. Moreover, information was collected on animal health, including vaccination, bedding and quarantine of purchased or sick animals. Finally, the interviewer’s observations regarding tick infestation on the animals and tick control methods used were recorded.

### 2.4. Statistical Analysis

The data collected from the farms were entered into Excel data sheets and pseudonymized, i.e., names and addresses of the farmers, farm owners and farm workers were removed and replaced by a unique identifier. These data were used for statistical analysis using R software version 4.0.4 and Rstudio version 1.4.1106 as an interface (R Core Team, 2021: Rstudio Core Team, 2021). Initially, the statistical relationship between the region and tick infestation was determined by Pearson’s chi-square test. Potential risk factors were tested against high tick infestation by univariable analysis using Fisher’s exact test. Variables like the presence of a quarantine area and the presence of another farm nearby were categorized as yes/no answers, whereas most of the other variables, i.e., type of bedding material (soft or hard), type of shed (open or closed), type of farm boundary (fence, wall or no boundary), drainage system on the farm (developed or not developed), waste disposal frequency (regularly or not regularly), vaccination of animals (vaccinated or non-vaccinated), visits by a veterinarian (regularly or not regularly), seasonal tick occurrence (summer or all seasons or summer/spring/autumn) and tick control treatment (acaricidal and/or herbal) were treated as categorical variables. 

For further analysis, a multivariable logistic regression model was used to determine any interacting effect of potential risk factors on tick infestation [24]. Variables (risk factors) with *p* < 0.2 in the univariable analysis were included in the multivariable analysis. The “Glm” function in R was used to run the model. Non-significant variables with *p* > 0.05 were removed from the model by stepwise backward elimination. Confounding effects were detected by observing changes in parameter estimates; if the change was more than 20% as compared to the full model, the variable was considered a confounder. The “vif” function was used to assess multicollinearity among the predictors. In the final model, the *p*-value, the odds ratio (OR), the regression coefficient and the ratio of the coefficient relative to the standard error were reported using the link function ‘logit‘. The 95% confidence intervals (CIs) were determined using the “exp” function. The Akaike information criterion was used to assess the model fit. The R-squared (R^2^) value was determined to assess the proportion of variability of the outcome variable that is explained by the predictors in the model.

## 3. Results

### 3.1. Characteristics of the Sampled Animal Population

In the 96 surveyed farms, the total number of farm animals was 2768, with a mean of 28.83 animals per farm and a median of 27.50 (Q1–Q3: 20.75–36; min–max: 12–57). Among these, 2583 (93.3%) were ruminants (cattle, buffaloes, sheep and goats), and 185 (6.7%) belonged to other species (dogs, donkeys, mules, horses, cats, rabbits and poultry) with a median of 2 (Q1–Q3: 1–2; min–max: 0–5). The majority of the ruminants were female (62.5%), with a median herd size of 5 in cattle (Q1–Q3: 3–7; min–max:1–12), 4 in buffaloes (Q1–Q3: 2–6; min–max: 2–12), 2.5 in sheep (Q1–Q3: 2–4.25; min–max: 0–9) and 3.8 in goats (Q1–Q3: 2–5; min–max: 0–12). Similarly, males comprised 968 (37.5%) of the ruminants with a mean herd size of 0.5 in cattle (Q1–Q3: 0–1; min–max: 0–6), 0 for buffaloes (Q1–Q3: 0–1; min–max:0–6), 3 in sheep (Q1–Q3: 2–5; min–max: 0–13) and 4 in goats (Q1–Q3: 2–6.25; min–max: 1–19). The 96 farms employed 360 workers, with a median of 3 (Q1–Q3: 3–5; min–max: 2–8), out of which 243 (67.5%) were males, 73 (20.3%) females, 24 (6.7%) children (under 10 years of age, without gender consideration) and 20 (5.6%) senior citizens.

### 3.2. Tick Infestation

**Figure 1:**
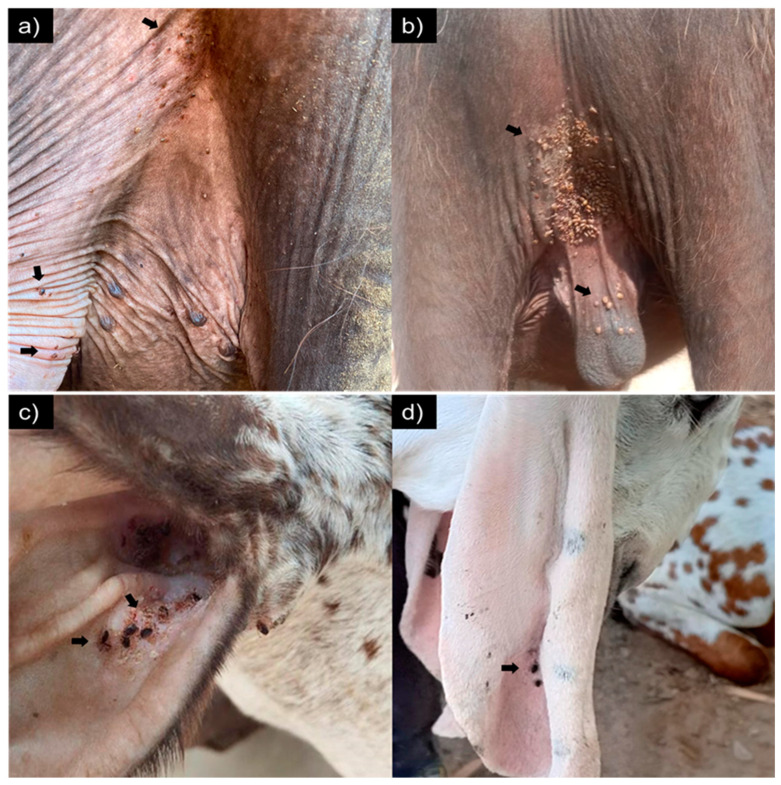
Tick infestation on the insides of the hindquarters of young buffaloes, (**a**) heifer and (**b**) bull, and on the surfaces of the ear of goats, (**c**) concave and (**d**) convex.

**Figure 2:**
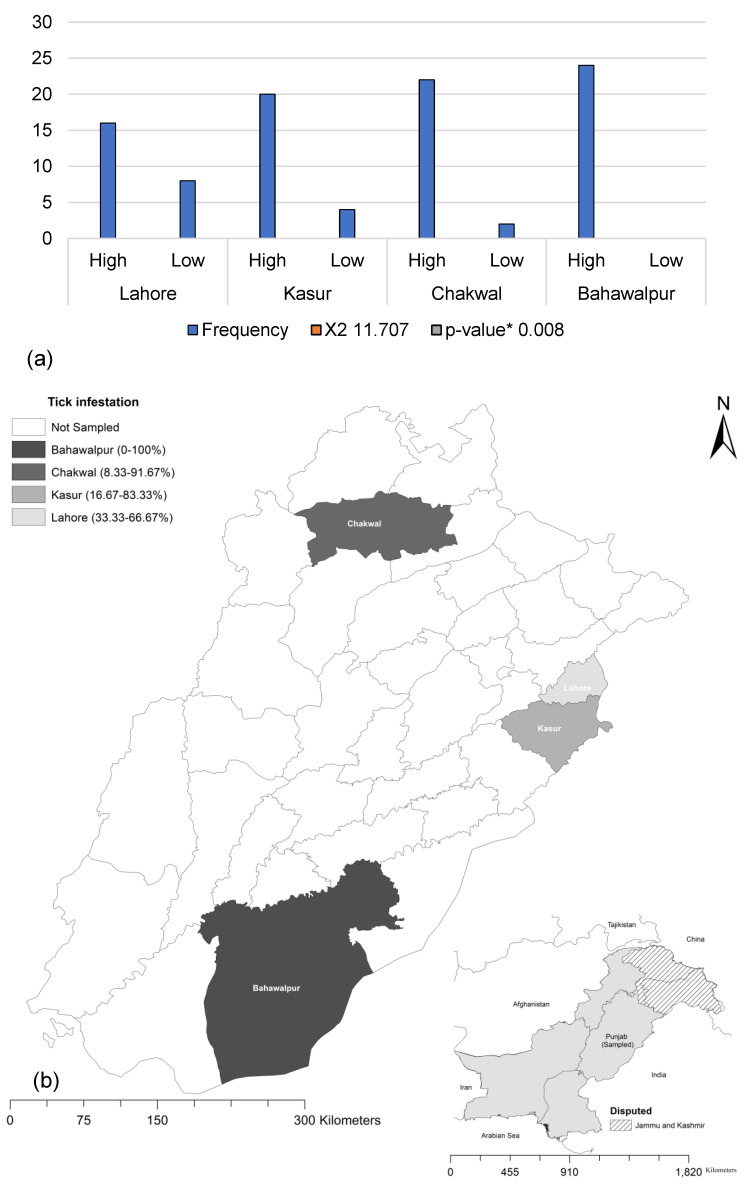
Pearson’s chi-square analysis of tick infestation; (**a**) across various districts; * *p* < 0.05 considered as significant (**b**) presented on map.

High tick infestation, defined as a minimum of five ticks per animal, was found in 85.4% (82/96) of farms (Figure 1). By location, Bahawalpur showed 100% (24/24) tick infestation, followed by Chakwal with 22 (91.7%), Kasur with 20 (83%) and Lahore with 16 (66.7%). A significant association (*p* < 0.05) between tick infestation and region was found using Pearson’s chi-square analysis (Figure 2.). The analysis showed a statistically significant association (*p* < 0.05) between tick infestation and location.

### 3.3. Identification of Potential Risk Factors by Univariable Analysis

The univariable analysis was conducted to identify factors contributing to tick infestation. Significant factors (*p* < 0.05) included the presence and duration of the quarantine area, the type of animal sheds, the type of farm bedding, drainage system efficiency, levels of animal morbidity and mortality, the frequency of veterinary visits, seasonal tick occurrence, tick treatment methods and the use of anti-tick drugs (Table 1). The study found that effective drainage systems, animal quarantine and tick treatment methods were statistically significantly associated with controlling tick infestation. Among the surveyed farms, 46.9% lacked adequate drainage and 18.8% of farms lacked any drainage system at all for cleaning and hygiene. Similarly, 52.1% of farms lacked a designated quarantine area, and 23.9% of the farm owners were unaware of quarantine importance. Of the 23 (23.9%) farms with quarantine areas, 34.8% quarantined their animals for 7 days, 43.5% for 14 days and 21.7% for 21 days.

Tick removal methods varied across the farms, with bathing of animals as the most common practice applied by 90 (93.7%) farms. In addition, 75 (78.1%) farms applied manual removal, 84.4% of farms used acaricidal drugs, 28.2% applied herbal medicine only and 12.5% both herbal and acaricidal drugs for the cases of very high tick infestation. A minority of the farms, i.e., 16.7%, used topical ointments to remove ticks from animals. As an anti-tick treatment, 56.3% of the farms used Ivermectin, 15.6% used Doramectin and another 15.6% applied aak plant (*Calotropis procera*) powder as a herbal remedy. The season was also statistically significantly (*p* < 0.0001) associated with tick infestation, with the highest figures in summer and spring together on 42 (43.7%) farms. Only 9 (9.4%) farms observed a high tick infestation ratio only in summer, but 23 (23.9%) farms reported tick-infested animals in every season (Supplementary Table S1). Pearson’s chi-squared analysis revealed a significant association of the region (district) in the study area with seasonal tick occurrence (chi-square value (*X*^2^*)* = 27.28, six degrees of freedom (df) and *p* = 0.01).

### 3.4. Multivariable Analysis

Out of the initial fifteen factors identified through univariable analysis for tick infestation, eleven variables with a *p* < 0.2 were selected for further analysis. Out of these, eight variables were incorporated into a multivariable logistic regression model. The variables “tick treatment” and “shed type” were excluded in favor of “anti-tick drugs” (correlation coefficient of 0.72) and “farm bedding” (correlation coefficient of 0.79), respectively. Finally, “seasonal tick occurrence” was also excluded due to the consistent presence of ticks on animals throughout the year, rendering it an inappropriate variable for this analysis. Henceforth, the initial model included seven categorical variables, i.e., quarantine area (*p* = 0.001), farm bedding (*p* < 0.001), drainage system (*p* < 0.001), morbidity (*p* < 0.001), mortality (*p* = 0.05), veterinary visits (*p* = 0.01), anti-tick drugs (*p* = 0.02) and one continuous variable—the total number of animals (*p* < 0.001). This model showed an R^2^ value of 0.6115 and an Akaike Information Criterion (AIC) of 52.98. Morbidity and mortality were excluded from the model, due to potential reverse causation, and the drainage system variable was replaced with the total number of animals (correlation coefficient 0.68). The final multivariable logistic regression model contained three variables, i.e., quarantine area, farm bedding and the total number of animals with an R^2^ value of 0.565 and an AIC of 42.658 (Table 2).

The variable ‘quarantine area’ (*p* = 0.191; 95% CI: 0.47–149.24) appeared to be non-significant, suggesting that it acted as a confounder, and its influence on tick infestation was either indirect or unclear. The model outcome indicated that larger herds tended to have a lower probability of tick infestation (OR = 0.87, coefficient estimate −0.13), possibly due to effective management practices in larger farms. In contrast, the use of soft farm bedding significantly increased the risk of tick occurrence (*p* = <0.003; 95% CI: 11.73–2557.44; OR = 100.88; and coefficient estimate 4.61), pointing to the critical role of bedding types in tick management.

## 4. Discussion

Tick infestation represents a major threat to livestock and human health around the globe. They not only impact the livestock economy due to their blood-sucking behavior but also due to their role as vectors in transmitting various bacterial, protozoal and viral pathogens. Tick infestation, especially in domestic livestock, is influenced by various factors such as host, geographical region and farm-related practices. Since tick prevalence is usually associated with farm practices and the environment, the objective of our study was to study the impact of farm-related practices on tick infestations in different agro-ecological zones. To this end, ninety-six farms were selected with a minimum of eight ruminants across four districts of Punjab, Pakistan. Pearson’s chi-square test and Fischer’s exact test were applied for regional and farm-related variables’ association. Finally, a multivariable analysis was performed to assess various risk factors using backward elimination.

Tick prevalence varied significantly across the districts in the univariable analysis, but not in the multivariable analysis. Recent studies in the districts of Lahore [8,25,26], Kasur [27,28], Chakwal [29,30,31] and Bahawalpur [29,31,32] have reported a high prevalence of tick infestations and tick-borne pathogens influenced by arid and semi-arid agro-ecological climates. This may be because Bahawalpur has a hot and dry climate, Chakwal has a local steppe climate, Kasur has a hot semi-arid climate and Lahore has a semi-arid climate bordering a humid subtropical climate [19].

In our study, most farms (76.04%) either lacked a quarantine area or did not practice it at all, which was initially significantly associated in the univariable analysis, but was not in the multivariable analysis. Although it did not come out as a risk factor in our study, previous studies have shown that quarantine combined with compulsory dipping and other preventive measures resulted in a rapid decrease in theileriosis in cattle, although the vector was not eradicated [33]. The farmers at these farms were aware of tick-borne diseases such as theileriosis, babesiosis and anaplasmosis, and reported cases of these diseases at their farms, which were treated by the veterinarians on duty. In this scenario, quarantine could be considered an important measure to minimize tick infestation as well as the occurrence of these diseases, as this period could be used for preventive measures against ticks, e.g., manual removing of ticks or dipping/applying acaricides to these animals.

Open types of sheds and a lack of developed drainage systems were associated significantly with tick infestation in the univariable analysis. This might be due to more suitable temperatures in open sheds and high humidity levels in inadequate drainage. Good ventilation and low humidity facilitated by a proper drainage system can help reduce tick burden on animals [34]. Another important finding in our study was that regular visits by veterinary staff were associated significantly with a lower prevalence of ticks at these farms. Although this association was not established in multivariable analysis, collaboration between farmers and veterinarians is essential for effective tick control in livestock [13,35].

Tick prevalence is significantly associated with seasonal variance, i.e., spring and summer in the univariable analysis, but not in the multivariable analysis. Although this study included districts from different agro-ecological zones of Punjab, overall, the province experiences extreme weather conditions with hot weather from April to June when the temperature rises up to 45–50 °C, especially in central and south Punjab, the monsoon season from July to September, and cold weather from October to March when the temperature falls to −10–2 °C, especially in north Punjab [36]. Such temperatures and humidity have been reported as risk factors for tick occurrence [8,37,38]. Since Pakistan is located in a sub-tropical region and ticks are more active in warm temperatures, tick infestations remain at a maximum in the spring and summer seasons of the country [8,37,38,39]. Tick infestation has posed significant economic losses on livestock farmers, especially in the areas where livestock farming is the main source of household income [13,39,40,41]. Such economic losses are not limited to decreased production but also anti-tick treatments and the morbidity and mortality of the animals due to tick-borne diseases [41,42].

Our study revealed a significant association between the use of acaricidal drugs and tick infestation in univariable analysis. The main drugs included cypermethrin and trichlorfon for external use [41,43]. Some farmers used traditional herbal remedies such as “Taramira Oil”, derived from roquette/rucola (*Eruca sativa*), in a solution of salt and water for bathing/drenching animals to control tick infestation [19,43]. Additionally, 28.12% of farmers used “Aak” powder made from *Calotropis procera* as a treatment for tick infestation, which was found effective against *Hyalomma* spp. ticks [44]. Avermectins, which are well-known drugs administered to treat worm infestations and external parasitic load on animals, were also used (Table 1). Ivermectin is one of the most potent drugs to tackle all stages of *Boophilus microplus* and sarcoptic mites [45,46]. Despite the use of both external and parenteral acaricidal drugs, ticks were still found on all surveyed farms in our study, which could be due to over-reliance on a single control method, leading to drug resistance. Previous studies have provided evidence of acaricidal drug resistance in ticks in Pakistan [43,47,48,49,50].

Bedding material, especially soft bedding composed of wheat straw, leaf litter or plain soil was significantly associated with the prevalence of tick infestation as compared to the hard bedding made up of concrete flooring. This appeared as a risk factor in the multivariable analysis, having a 100 times increased risk of tick infestation. A study in Portugal indicated a minimum risk of tick infestation on animals by not using bedding material at all, regularly washing the floor and regularly monitoring the animals [51]. Another study in Bhutan indicated a possible association between the use of leaf litter as bedding material and tick infestation in cattle. In the same study, farmers considered using bracken fern as bedding material as one of the sources of tick infestation in their cattle sheds [52]. Previous studies indicated that traditional rural housing (structures made of mud bricks and wood) and inadequate farm structures such as poor ventilation and drainage systems coupled with improper sanitation increased the tick infestation risks compared to those of commercial and developed farms [53]. The study also found that the majority of farms (77.1%) used soft bedding, underscoring the need for a re-evaluation of husbandry practices on these farms.

There were certain limitations in our study, e.g., the study focused on selected districts in Punjab, which could limit general predictions across the regions where climate conditions and farming practices are different. Moreover, being a cross-sectional study, it captured data at a single point in time, specifically the winter season, which could potentially limit a full understanding of how various factors would be associated with the tick infestation rates throughout the year. The reliability of the data may be influenced by factors such as the respondents’ willingness to participate and knowledge and accuracy of the provided information [54]. Despite these limitations, this study provided valuable insights into the factors influencing tick infestation, e.g., location, farm management, husbandry practices and the effects of season and climate on livestock. This could contribute to a broader understanding of tick management challenges and needs globally, especially in the surrounding districts and even neighboring countries. There is a critical need for enhanced education and awareness about effective tick management and resistance prevention among livestock farmers. This calls for coordinated efforts from the livestock and public health departments of Punjab, Pakistan to implement tick control strategies to control tick-borne pathogens in animals and prevent zoonotic transmission.

## 5. Conclusions

This study highlighted various management factors, e.g., absence of quarantine, shed types, bedding, drainage, veterinary services, use of acaricides and seasonal variations that influenced tick infestation on these farms. Soft bedding significantly increased the tick infestation risk, suggesting a transition towards hard bedding materials such as concrete floors. Establishment of quarantine practices, when introducing new animals, along with improved farm structures, focusing on ventilation and drainage systems, should be applied. Farmers should apply a combination of chemical and traditional remedies to tackle drug resistance in ticks. Regular inspection of animals by the veterinary staff and maintenance of these measures by the farmers are necessary. Education and awareness-raising programs for the farmers on tick-borne diseases and their transmission are needed. Further research is encouraged to identify tick populations and tick infestation dynamics through longitudinal studies, considering host factors and the development of effective acaricides to tackle resistance in ticks. Joint tick control and awareness programs need to be implemented involving farmers, veterinarians, research institutions and medical health organizations.

## Figures and Tables

**Table 1:** Fisher’s exact test analysis of the variables influencing high tick infestation at the farms.

Variable	Category	No. of Responses	*p*-Value
Quarantine area	Present	23	<0.001
	Not Present	73	
Quarantine period (days)	0	73	<0.001
	7	8	
	14	10	
	21	5	
Shed type	Open	75	<0.001
	Closed	21	
Farm bedding	Soft	74	<0.001
	Hard	22	
Nearby farm (500 m radius)	Present	63	0.547
	Not Present	33	
Farm boundaries	Present	95	1
	Not Present	1	
Type of farm boundary	Fences	12	0.319
	Concrete/Mud	83	
	No Wall	1	
Drainage system	Developed	33	<0.001
	Not Developed	63	
Waste disposal frequency	Regularly	90	0.588
	Not Regularly	6	
Frequency of veterinary visits	Regularly	30	0.015
	Not Regularly or No Visit	66	
Vaccination status	Vaccinated	79	1
	Non-Vaccinated	17	
Seasonal tick occurrence	Summer	9	<0.001
	Summer/Spring (Both)	42	
	Summer/Spring/Autumn	22	
	Every Season	23	
Tick treatment	Acaricidal	69	0.036
	Herbal	15	
	Acaricidal/Herbal (Both)	12	
Anti-tick drugs (allopathic and herbal)	Aak Pulv (*C. procera*)	15	0.023
	Ivermectin	54	
	Doramectin	15	
	Ivermectin/Aak (Both)	12	

**Table 2:** Results of final multivariable logistic regression model.

Variable	Estimate	Odds Ratio	Standard Error	95% Confidence Interval	*p*-Value
Quarantine Area	1.86	6.47	1.43	0.47–149.24	0.191
Farm Bedding	4.61	100.88	1.28	11.73–2557.44	<0.003
Total Animals	−0.13	0.87	0.06	0.76–0.98	0.03

R^2^ = 0.565; AIC = 42.658.

## Data Availability

Data can be provided upon request.

## Supplementary Materials

The following supporting information can be downloaded at https://www.mdpi.com/article/10.3390/ani14162437/s1.
