# Supplementary material for: Impact of Farm Management Practices on Tick Infestation in Punjab’s Livestock: A Comprehensive Epidemiological Study
Source: Animals (Basel). 2024 Aug 22;14(16):2437. doi: 10.3390/ani14162437 (PMC11350798; doi:10.3390/ani14162437)
Supplement: Supplementary file 1 [file animals-14-02437-s001.zip › animals-3024229-supplementary.pdf]

Supplementary Table S1: Responses and Frequencies of answers from livestock owners/managers

Sr. No	Question	Response	Frequency	Percentage
1[a]	Is there a quarantine area specified for animals at farm?	Present	23	23.96
		Not Present	50	52.08
		No Idea	23	23.96
2	What is the minimum quarantine period for animals at farm?	0 days	73	76.04
		7 days	8	8.33
		14 days	10	10.42
		21 days	5	5.21
3	What type of shed (housing) is provided at farm?	Open	75	78.13
		Closed	21	21.87
4	Which kind of bedding for animals is provided at farm?	Soft[1]	74	77.08
		Hard[2]	22	22.92
5	Is there any other farm nearby in 500 m radius?	Present	63	65.63
		Not Present	33	34.37
6	Does the farm have any boundaries?	Present	95	98.96
		Not Present	1	1.04
7	Is there any farm boundary present around peripheries of farm for animals? If yes, what kind of?	Fences	12	12.5
		Concrete/Mud	83	86.46
		No Wall	1	1.04
8[b]	Has the farm drainage system for cleanliness? If yes, how is the system?	Developed[3]	33	34.38
		Not Developed[4]	45	46.88
		No system	18	18.75
9[c]	What is the waste disposal frequency at farm?	Daily	90	93.75
		Weekly	6	6.25
		Month	0	0
10[f]	How often is the farm visited by a veterinarian?	Daily	4	4.17
		Weekly	26	27.08
		Month	59	61.46
		No Visit	7	7.29

11	What is the vaccination status of animals at the farm?	Vaccinated[11]	79	82.30
		Non-Vaccinated	17	17.70
12[g]	What is the tick infestation frequency at farm level	High[12]	82	85.42
		Medium[13]	13	13.54
		Low[14]	1	1.04
13	In which season do tick infestations occur at farm?	Summer	9	9.38
		Summer/Spring (both)	42	43.75
		Summer/Spring/Autumn	22	22.92
		Every season[15]	23	23.95
14	Which common anti-tick protocol is used at farm for tickremoval?	Hand pick removal	75	78.12
		Bathing of animal	90	93.75
		Application of ointments	16	16.67
		Use of herbal medicine	27	28.17
		Use of allopathicmedicine	81	84.38
15	What is the common tick treatment used at farm?	Acaricidal	69	71.88
		Herbal	15	15.62
		Acaricidal/Herbal (both)	12	12.50
16	Which anti-tick drugs (Allopathic and herbal) are usedat farm?	Aak Pulv (*C. procera*)	15	15.63
		Ivermectin	54	56.25
		Doramectin	15	15.62
		Ivermectin/Aak (Both)	12	12.5

[1]Soft Bedding (Mud, wheat straw and leaf litter or anyone of these),[2]Hard Bedding (Concrete Floor),[3]Developed (This consist of pipelines for sewage and water supply within Farm and connected to main public sewage, manholes and waste water lines),[4]Not Developed (The system for sewage and waste water within farm with blockage, leakage and overflow in the pipelines and manholes together with incomplete construction or any of these), [11]Vaccinated Animals (All the ruminants at farms that were inoculated with vaccines during the vaccination campaigns of Livestock and Dairy Development Department Govt. of Punjab before the sampling period) [12]high (5 and more ticks observed on animal body),[13]medium (2-4 ticks observed animal body), [14]low (0-1 ticks observed on animal body), [15]Every Season (It includes Spring, Winter, Summer, Autumn)

[a] The response in question 1. Categories ‘Not Present’ and ‘No Idea’ were combined to category ‘Not-Present’ for the analysis.
[b] The response in question 8. ‘Not properly developed’ and ‘No system’ were combined to category ‘Not-Developed’ for the analysis
[c] The response in question 9. Category ‘Daily’ refers to the category ‘Regularly’; Weekly and Monthly were combined to category ‘Not-regularly’ for the analysis.
[d] The response in question 10. Low and medium categories were combined to category ‘Low’ for the analysis
[e] The response in question 11. High and medium categories were combined to category ‘High’ for the analysis.
[f] The response in question 12. Daily and Weekly categories were combined to ‘Regularly’ for the analysis; Monthly or No visit categories were combined ‘Not Regularly’ category for analysis.
[g] The response in question 14. Low and medium categories were combined to category ‘Low Tick Infestation’ for the analysis
